# Model Studies on the Photoreduction of the 5‐Hydroxy‐5,6‐dihydrothymine and 5‐Methyl‐2‐pyrimidone Moieties of (6‐4) Photoproducts by Photolyase^†^

**DOI:** 10.1111/php.13592

**Published:** 2022-01-28

**Authors:** Gemma M. Rodríguez‐Muñiz, Miguel A. Miranda, Virginie Lhiaubet‐Vallet

**Affiliations:** ^1^ Instituto Universitario Mixto de Tecnología Química (UPV‐CSIC) Universitat Politècnica de València Consejo Superior de Investigaciones Científicas Valencia Spain

## Abstract

Photorepair mechanism of (6‐4) photoproducts (6‐4PP) by photolyase has been the subject of active debate over the years. The initial rationalization based on electron transfer to an oxetane or azetidine intermediate formed upon binding to the enzyme has been questioned, and there is now a more general consensus that the lesion is directly reduced from the excited flavin cofactor. However, the accepting moiety, i.e. the 5‐methyl‐2‐pyrimidone or 5‐hydroxy‐5,6‐dihydrothymine, has not been fully identified yet. In this work, spectroscopic experiments have been run to determine which of the 5′‐ or 3′‐base of 6‐4PP is more prone to be reduced. For this aim, the two building blocks of 6‐4PP were synthesized and used as electron acceptors. Instead of the short‐lived photolyase cofactor, which does not provide a time window compatible with diffusion‐controlled intermolecular processes, carbazole, 2‐methoxynaphthalene and phenanthrene have been selected as electron donors due to their appropriate singlet lifetimes and reduction potentials. Steady‐state and time‐resolved fluorescence revealed that, in solution, the pyrimidone chromophore is the most easily reduced moiety. This was confirmed by transient absorption experiments consisting of quenching of the solvated electron by the two moieties of 6‐4PP.

## Graphical abstract

Which is the electron accepting moiety during (6‐4) photoproducts repair by photolyase? A model study using 5‐hydroxy‐5,6‐dihydrothymine and 5‐methyl‐2‐pyrimidone.

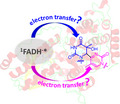

## INTRODUCTION

Sunlight is the main etiological cause of photocarcinogenesis as it contributes to the development of different skin cancers such as squamous cell or basal cell carcinoma and melanoma (1, 2, 3, 4). In this respect, it has been classified, together with its UV components, as a “class I carcinogen” by the *International Agency for Research on Cancer* (IARC) (5). From a chemical point of view, UV radiation is able to modify the integrity of the DNA molecule by mediating pyrimidine (Pyr) dimerization, which leads to the formation of cyclobutane pyrimidine dimers (CPD) and (6‐4) photoproducts (6‐4PP) as the main direct damages (Chart 1) (6, 7). The role of these lesions in UV mutagenesis has been clearly established by the coincidence of hotspots for their formation and the occurrence of mutations (8, 9, 10, 11, 12).

**Chart Chart 1:**
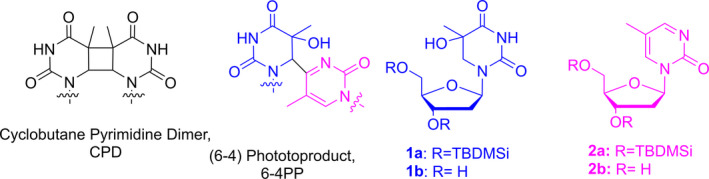
Structure of 6‐4PP and of its building blocks **1** and **2**.

Fortunately, our organism has an efficient DNA repair machinery that allows preservation of the genome integrity in spite of its continuous exposure to damaging agents. The importance of this machinery has been highlighted by the Nobel Prize in Chemistry 2015 awarded jointly to Lindahl, Modrich and Sancar for “mapping the fundamental processes of DNA repair at the molecular level” (13). In mammals, CPD and 6‐4PP are removed by the nucleotide excision repair (NER) system. The relevance of this pathway for human health is manifested by the severe clinical consequences associated with inherited NER defects linked to diseases such as Xeroderma pigmentosum (XP), whose patients are exposed to an increased risk to sunlight‐induced skin cancer by respect with non XP patients (6).

Remarkably, the discovery in other organisms of an additional repair pathway for Pyr dimers has aroused the interest of scientists to develop alternative strategies for artificial DNA repair in humans. This process known as DNA photoreactivation is catalyzed by peculiar enzymes that take advantage of solar light to trigger the photoreaction. These enzymes, called photolyases, consist in a single polypeptide chain with two prosthetic groups: a pterin molecule in the form of methenyltetrahydrofolate (MTHF) and a fully reduced deprotonated flavin molecule (FADH^‐^) (14, 15). The former acts as a light harvesting photoantenna, which after absorption of a photon transfers its energy to the catalytic FADH^‐^ cofactor. Then, in the case of CPDs, for which the photorepair mechanism is well‐established (14, 16, 17), the singlet excited flavin (^1^FADH^‐*^) donates an electron to the ground state lesion and subsequent ring splitting of the cyclobutane radical anion restores the native Pyr nucleobases. By contrast, photorepair of 6‐4PP entails an additional difficulty compared to the CPD case because restoration of the initial Pyr sequences requires the back transfer of the 5′‐side hydroxyl (or amino group) to the 3′‐base. This explains why this mechanism has been the subject of active debate and is still not fully resolved (16, 17, 18, 19, 20, 21, 22).

**Scheme 1:**
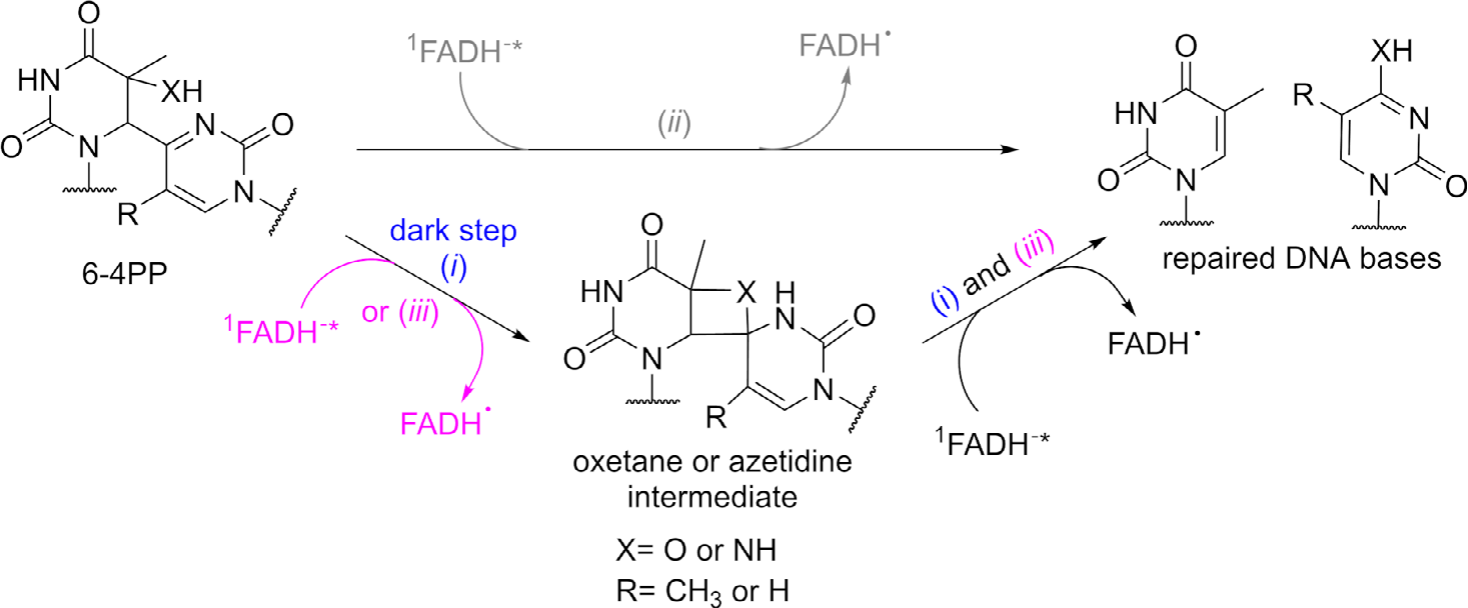
Proposed mechanisms for 6‐4PP repair by photolyase.

For a longtime, it was accepted that the (6‐4) photolyase operates through an intramolecular dark rearrangement of the lesion to form an oxetane (or azetidine) intermediate, whose subsequent reduction triggers the cycloreversion and repair process (Scheme 1, pathway *i*) (15). However, this mechanism has been challenged by an *in situ* study of the crystallized photolyase containing a single lesion (19, 20). The lesion was shown to maintain its structure upon binding to the enzyme, which discards the formation of an oxetane in a dark step. In addition, photorepair within the crystal without strong variation of the substrate surroundings led to the conclusion of a direct photoinduced electron transfer from ^1^FADH^‐*^ to 6‐4PP (Scheme 1, pathway *ii*). Nonetheless, another proposal based on kinetic and theoretical studies has revived the oxetane/azetidine scenario. It consists in a two‐photon repair process during which the initial photogeneration of the heterocyclic intermediate is followed by a photoinduced electron transfer (Scheme 1, pathway *iii*) (22). It is noteworthy that, in the abovementioned mechanistic schemes, two conserved histidines were revealed to be crucial for the photorepair process and were proposed to play the role of an acid–base catalyst for the oxetane/azetidine intermediate formation or for the migration of the hydroxy group from the 5′ to the 3′ nucleobase (17, 23).

Repair of 6‐4PP also differs from that of CPD in terms of quantum yields (ϕ). CPD are efficiently restored by photolyases with ϕ as high as 40–100%, whereas values for 6‐4PP are between 4 and 100 times lower (17). Ultrafast spectroscopy associated this difference with a futile back‐electron transfer from the lesion anion radical to the semireduced FADH^•^. This process, one or two order of magnitude faster in the case of 6‐4PP photolyases, competes with the subsequent steps of the repair pathway and leads back to FADH^‐^ and to the lesion in its initial form (16, 17).

Interestingly, the electron accepting moiety of 6‐4PP, i.e., the 5‐methyl‐2‐pyrimidone or 5‐hydroxy (or amino)‐5,6‐dihydrothymine, has not been established yet; actually, both substructures have been proposed to be reduced firstly, depending on the experimental or theoretical study (16, 17, 24, 25). Here, spectroscopic experiments were carried out to determine which of the 5′ or 3′ moieties of 6‐4PP is more prone to be reduced. In this context, two models of the (6‐4) photolesion were synthesized (compounds **1** and **2**, Chart 1) and used as potential electron acceptors for steady‐state and time‐resolved studies.

## MATERIALS AND METHODS

### Chemicals

All chemicals were obtained from commercial sources and were used without further purification. Nuclear magnetic resonance (NMR) spectra were performed on a Bruker Avance 400 MHz.

### Synthesis

5‐Methyl‐2‐pyrimidinone derivatives (Chart 1, **1a** and **1b**) were prepared as previously reported (26). Briefly, 3′ and 5′ hydroxyl groups of thymidine were first protected as tertbutyldimethylsilyl (TBDMSi) group, the obtained compound was then treated with P(O)Cl_3_/triazole to give quantitatively the 4‐(triazol‐1‐yl) derivative. Thereafter, treatment with fresh anhydrous hydrazine gave the hydrazino intermediate, which was not isolated and was immediately oxidized with Ag_2_O to obtain **1a**. Finally, **1b** was formed after deprotection of the TBDMSi groups using tetrabutylammonium fluoride (TBAF). Compounds **2a** and **2b** were synthesized following the previously described methodology (27). Briefly, dihydroxylation of disilylated thymidine was achieved using catalytic osmium tetroxide and N‐methylmorpholine. The secondary alcohol was converted into a benzoate, and **2a** was obtained after a photochemical method introduced by Saito *et al* (28). Desilylation to yield **2b** was performed using TBAF.

### UV‐Vis absorption

Absorption spectra were recorded on a Cary 60 spectrometer by using quartz cuvettes with 1 cm optical path lengths.

### Fluorescence

Fluorescence measurements were carried out on a with a FLS1000 fluorometer (Edinburgh instruments), equipped with a PMT‐980 detector. Excitation was afforded by a 450W Xe lamp or EPLED‐260 Diode (*λ*_exc_ = 256.6 nm) for steady‐state or time‐resolved experiments, respectively. All experiments were run under air in a 1 cm optical path length quartz cuvette, and the absorbance of the photoreductant was kept under 0.15 at the excitation wavelength in acetonitrile (*λ*_exc_ = 260 nm). Concentrations and molar absorption coefficients of **1a**, **2a** and photoreductants are given in Table S1. Stock 0.14 M solutions of the quenchers (Q), i.e. **1a** or **2a**, were prepared in acetonitrile, so it was only necessary to add microliter volumes to the sample to obtain the final concentrations of the quencher.

For steady‐state experiments, the bimolecular rate constants, *k*_q_(SS), were obtained from the Stern–Volmer plots by using Equation (1):

(1) $$I_{0}/I=1+K_{\text{SV}}\times \left[Q\right]$$

in which *I*_0_ and *I* are the emission intensity of the PR in the absence of Q and after the addition of a quencher concentration [*Q*], respectively; *K*_SV_ (*K*_SV_= *k*_q_(SS) × *τ*_0_) is the Stern–Volmer rate constant obtained from the slope and τ_0_ is the lifetime of the photoreductant in the absence of *Q*.

For time‐resolved fluorescence experiments, the kinetic traces were fitted by a mono‐exponential decay function, by using a deconvolution procedure to separate them from the lamp pulse profile.

The rate constants *k*_q_(TR) for the reaction were obtained from the Stern–Volmer plots by using Equation (2):

(2) $$1/t=1/t_{0}+k_{q}\left(\mathrm{TR}\right)\times \left[Q\right]$$

in which *τ* is the lifetime of the photoreductant in the presence of the quencher at a given concentration [*Q*].

### Laser flash photolysis

Laser flash photolysis experiments were performed exciting at 266 nm, using the 4^th^ harmonic of a pulsed Nd:YAG laser (L52137V LOTIS TII) with a pulse duration of 6‐8 ns; an intensity of 10 mJ/pulse was used. The full system consists in a pulsed laser, a Xenon lamp (Lo 255 Oriel), a monochromator (Oriel 77200), a photomultiplier (Oriel 70705) and an oscilloscope (TDS‐640A Tektronic). The output signal from the oscilloscope was transferred to a personal computer. All experiments were performed in a quartz cell, with screw cap and septum, of 1 cm optical path length and deaerated with N_2_. *N,N*‐Dimethylaniline (DMA, 5.4 × 10^‐5 ^M) solution was prepared in water to obtain an absorbance of ca. 0.4 at 266 nm after bubbling with N_2_. Stock 2.85 mM solutions of **1b** or **2b** were prepared in water. The rate constants *k*_qe_ for quenching of the hydrated electron were obtained from the Stern–Volmer plots, using equation (2), where this time *k*_qe_ = *k*_q_(TR), *τ*_0_ and *τ* are the lifetimes of the hydrated electron in the absence or in the presence of quencher at a concentration [*Q*].

## RESULTS

### Steady‐state and time‐resolved fluorescence

The efficiency of the electron‐transfer process from photoreductants (PR) to **1a** or **2a** was determined by steady‐state (SS) and time‐resolved (TR) fluorescence. Monitoring the changes in the intensity and/or kinetics of ^1^FADH^‐*^ emission in the presence of these compounds would, in principle, inform on the electron transfer toward one or another of the 6‐4PP moieties. However, the very short lifetime of the photolyase cofactor (in the picosecond timescale) does not provide a time‐window compatible with diffusion‐controlled intermolecular reaction. (14) Thus, to overcome this limitation and best reproduce the natural photolyase cofactor activity, PR were selected on the basis of their reduction potential, to be close to that of ^1^FADH^‐*^(*E*_red_* of ca. −2.9 V vs SCE), (29) but also for their singlet lifetime decaying in the nanosecond timescale (Table 1) (30, 31, 32).

**Table 1:** Fluorescence lifetime and reduction potential in the singlet excited state (*E*_red_*) of the selected photoreductants and FADH^‐^ in their singlet excited state.

Photoreductant, PR	Lifetimes	E*_red_ in SCE
FADH^‐^	68 ps^a^	−2.9 V^b^
Carbazole	7.6 ns	−2.5 V^c^
2‐Methoxynaphthalene	7.5 ns	−2.3 V^d^
Phenanthrene	12.9 ns	−2.1 V^d^

^a^Mean lifetime from ref (14); ^b^from ref (29); ^c^ from ref (31); ^d^ from ref (32).

**Figure 1:**
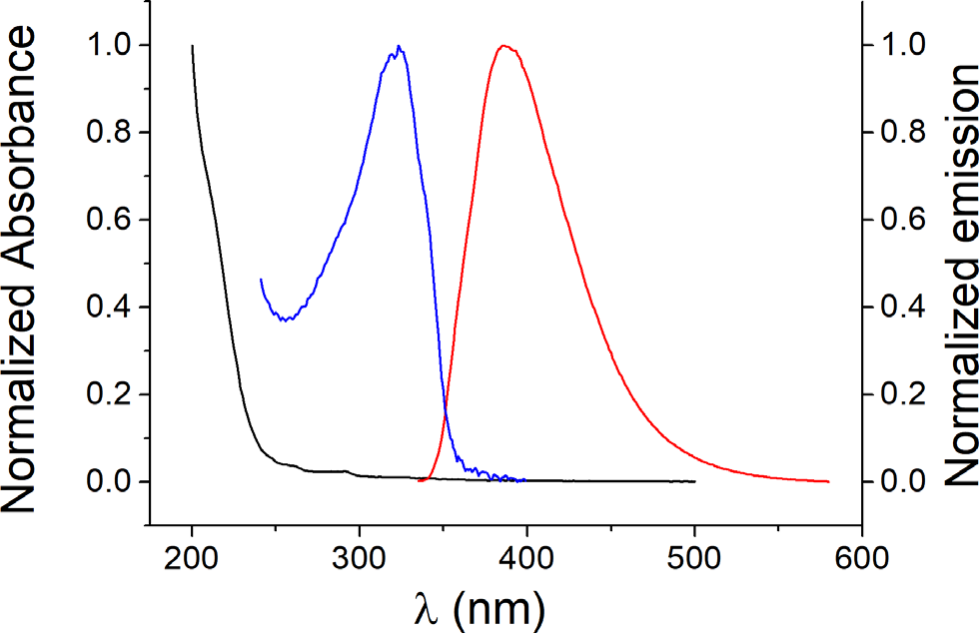
Normalized absorption spectra of **1a** (black) and **2a** (blue) in acetonitrile, and normalized emission spectrum of **2a** (red) after 260 nm excitation.

In a first stage, steady‐state fluorescence experiments were run. Solutions of the selected PR were prepared in acetonitrile, and their emission spectra were registered in the absence and in the presence of **1a** or **2a** (Chart 1), which were used as quenchers. The inherent absorption and emission of 2‐pyrimidone chromophore (Fig. 1) (33, 34) limit the choice of the excitation wavelength (*λ*_exc_) to excite selectively the PR. A *λ*_exc_ of 260 nm, where all the PR have a higher molar absorption coefficient than **2a** (Table S1), was finally selected as the best option to minimize direct excitation of the quencher. The same *λ*_exc_ was used for quenching experiments with **1a** as it does not exhibit any absorption in the UV range (Fig. 1).

**Figure 2:**
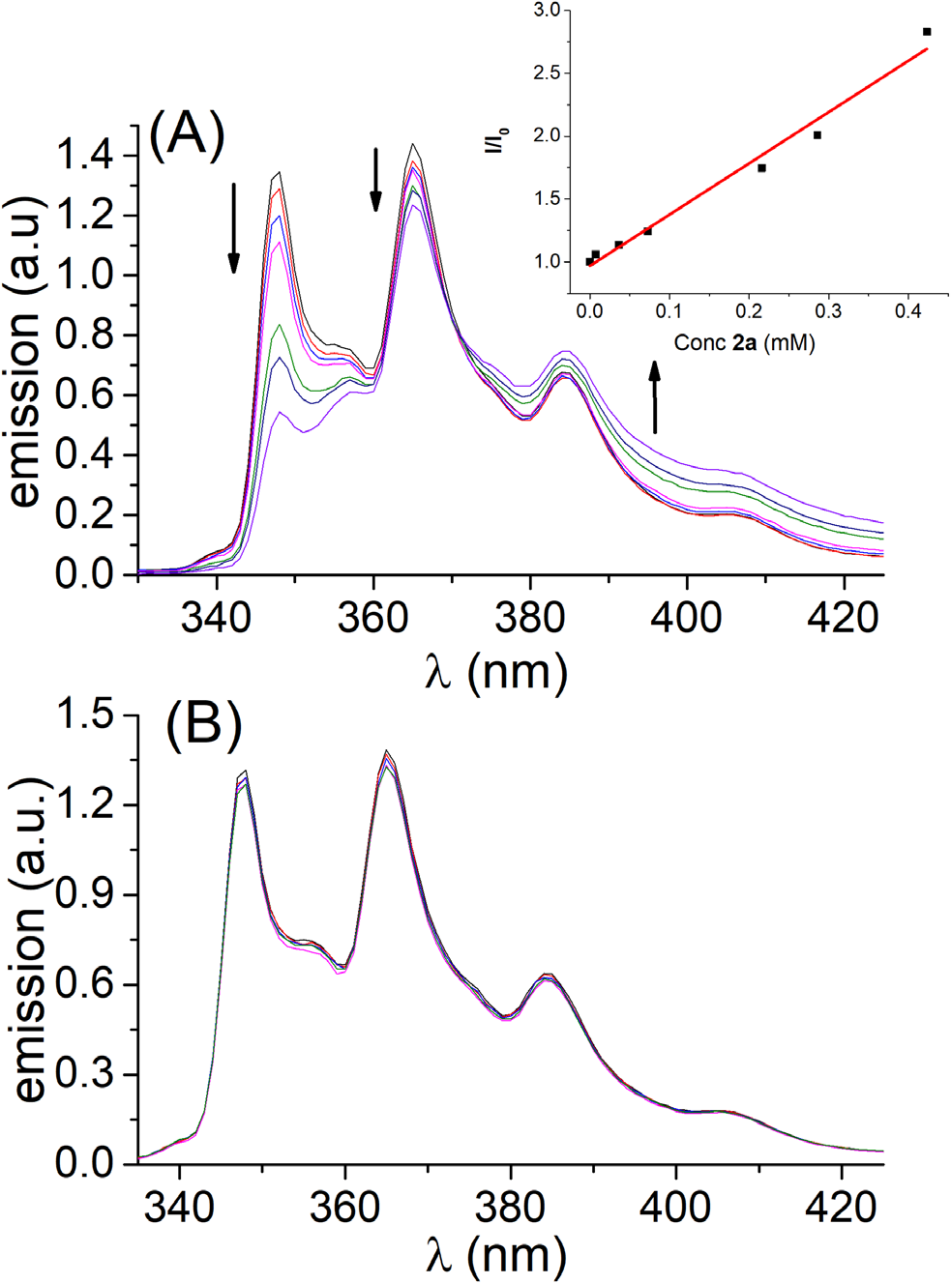
Steady‐state fluorescence spectra of phenanthrene in acetonitrile (λ_exc_=260 nm) in the presence of increasing amounts (up to 0.42 mm) of **2a** (A) or **1a** (B).

Figure 2 shows the evolution of phenanthrene emission in the presence of increasing amounts of both quenchers. In the presence of **1a**, the fluorescence intensity did not exhibit significant variation, whereas addition of **2a** resulted in important and uneven changes of the emission spectra. The 0‐0 band at 347 nm vanishes with increasing **2a** concentration, whereas the band at 365 nm decreases to a much lower extent. In parallel, the emission intensity grows at the red side of the spectra (at *λ* > 370 nm). This behavior can be explained by a screening effect due to the spectral overlap between the emission of the donor, namely phenanthrene, and absorption of the acceptor, **2a** (see Figure S1). Moreover, although minimized at 260 nm, the direct absorption of **2a** cannot be avoided and leads to its intrinsic fluorescence emission with a maximum wavelength ca. 400–410 nm (Figure S2). Changes of the 365 nm band might be attributed to an energy or electron transfer process. However, the former can be discarded as the singlet excited state energy of **2a**, E_S_=351 kJ mol^‐1^ (34), is higher than that of phenanthrene of ca. 346 kJ mol^‐1^ (35). Therefore, bimolecular rate constant corresponding to the electron‐transfer process, *k*_q_(SS), was determined as the slope of the regression line obtained from the Stern–Volmer plot (see eq. (1) in the Material and Methods section, and inset in Fig. 2A), which represents the variation of the fluorescence intensity (*I*/*I*_0_) as a function of the quencher concentration. The presence of the screening effect and direct absorption by **2a** hampers an accurate calculation of the *k*_q_(SS) value because these processes have a strong contribution in the changes in the intensity of the 347 and 385 nm bands. Thus, the intensity at 365 nm was selected to represent the Stern Volmer plot, and an upper limit *k*_q_(SS) value of 6.9 × 10^10^ M^‐1^s^‐1^ was estimated for the electron transfer process between phenanthrene in its singlet excited state and **2a**.

This behavior was observed for the quenching by the nonabsorbing compound **1a**, where a very weak decrease of emission was monitored (Fig. 2B). Similar results were observed for the combination of the other two photoreductants, carbazole and 2‐methoxynaphthalene, with the two moieties of 6‐4PP (see Figure S3).

**Figure 3:**
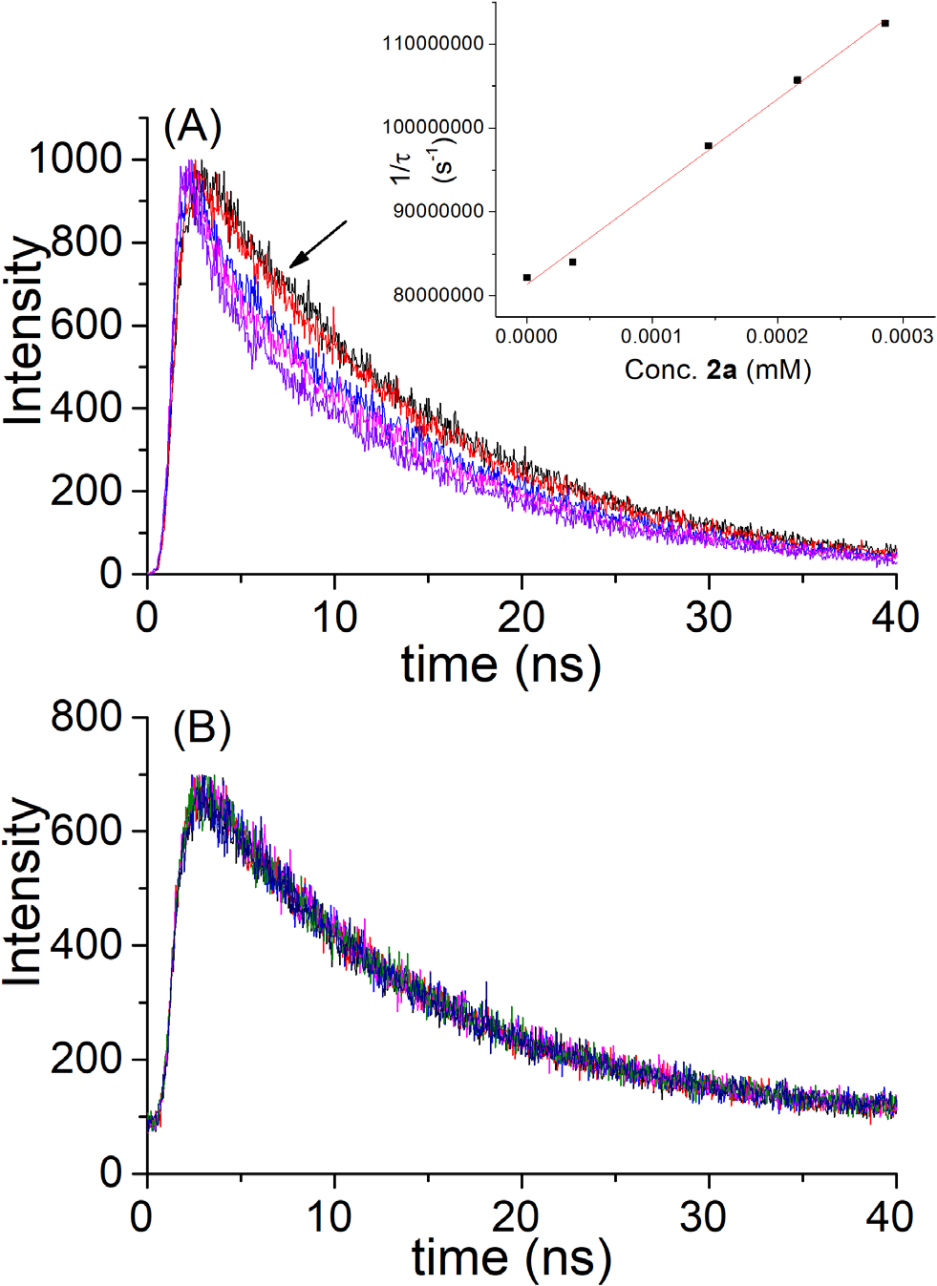
Kinetic traces of phenanthrene in acetonitrile (λ_exc_=256 nm, λ_em_=371 nm) in the presence of increasing amounts (up to 0.36 mm) of **2a** (A) or **1a** (B).

In the next stage, time‐resolved fluorescence studies were performed to evaluate whether dynamic quenching was taking place. Indeed, screening effect does not affect the lifetime of the donor, thus more accurate values for the electron transfer process might be obtained. As in the case of steady‐state experiments, an excitation wavelength close to 260 nm (using a diode emitting at 256.6 nm) was selected to minimize direct excitation of **2a**, and detection was performed at 371 nm where this fluorophore exhibits a low emission. As shown in Fig. 3A, a shortening of the phenanthrene lifetime was observed after addition of **2a**. The decays were fitted with the monoexponential function *f*(*t*)=A exp(‐*t*/*τ*) for **2a** concentration up to 0.36 mM, but at higher concentration values a short‐lived component, with a lifetime of 1.5 ns, appeared. It was attributed to the contribution of **2a** fluorescence produced by direct excitation (Figure S2). Thus, the measurements were limited to low quencher concentrations, where **2a** lifetime does not contribute to the kinetics. Under these conditions, a diffusion‐controlled bimolecular rate constant, *k*_q_(TR), of *ca*. 6.8 × 10^10^ M^‐1^s^‐1^ was determined for the electron‐transfer process using eq. (2) (see Material and Methods section). By contrast, and as expected from the steady‐state fluorescence, no dynamic quenching by **1a** was observed (Fig. 3B).

Hence, both steady‐state and time‐resolved results pointed towards a more efficient electron transfer to **2a**. However, the absorption and fluorescence properties of the pyrimidone chromophore limit the study, and its extension to other PR (see Supporting Information, Figure S3).

### Laser flash photolysis

**Figure 4:**
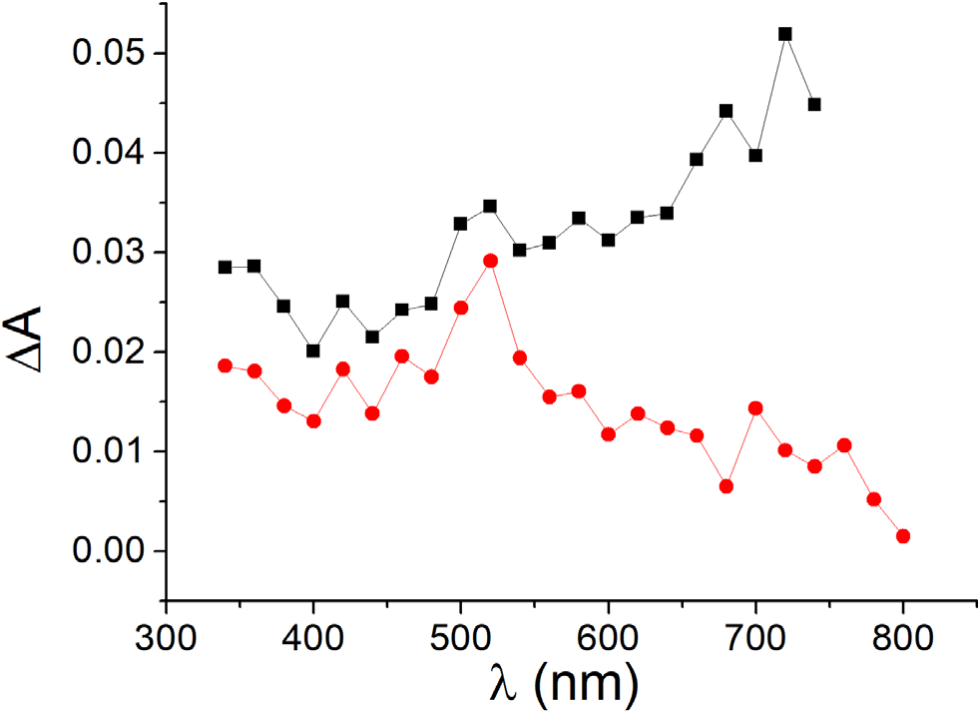
Transient absorption spectra of *N*,*N*‐dimethylaniline (A_266_=0.4) in water 0.14 μs after the 266 nm laser pulse under N_2_ (black line) or N_2_O atmosphere (red line).

Thus, a different approach was designed to circumvent the difficulties associated with the pyrimidone chromophore fluorescence. This approach, based on transient absorption spectroscopy, allows comparing the ability of the 6‐4PP moieties to act as electron acceptors. For this purpose, hydrated electron (e^‐^_aq_) was generated from photoionization under 266 nm laser excitation of *N,N*‐dimethylaniline (DMA) in water. This species exhibits a typical transient absorption with a maximum at 720 nm, where no interference is expected from the 2‐pyrimidone chromophore (33). Thus, monitoring of the e^‐^_aq_ kinetics as a function of the 6‐4PP components concentration will reveal their ability to accept an electron, and thus, comparison of the quenching rate constants will inform on the moiety which is more prone to be reduced during the photorepair process. In this context, nitrogen‐bubbled water solution of DMA was excited at 266 nm using the 4^th^ harmonic of a Nd:YAG laser. The transient absorption spectrum showed the characteristic broad signal of the hydrated electron, with a band maximum at 720 nm and a lifetime of ca. 1.6 μs (Fig. 4). As expected, this signal was efficiently quenched when the solution was bubbled with N_2_O (Fig. 4, red line) (36).

**Figure 5:**
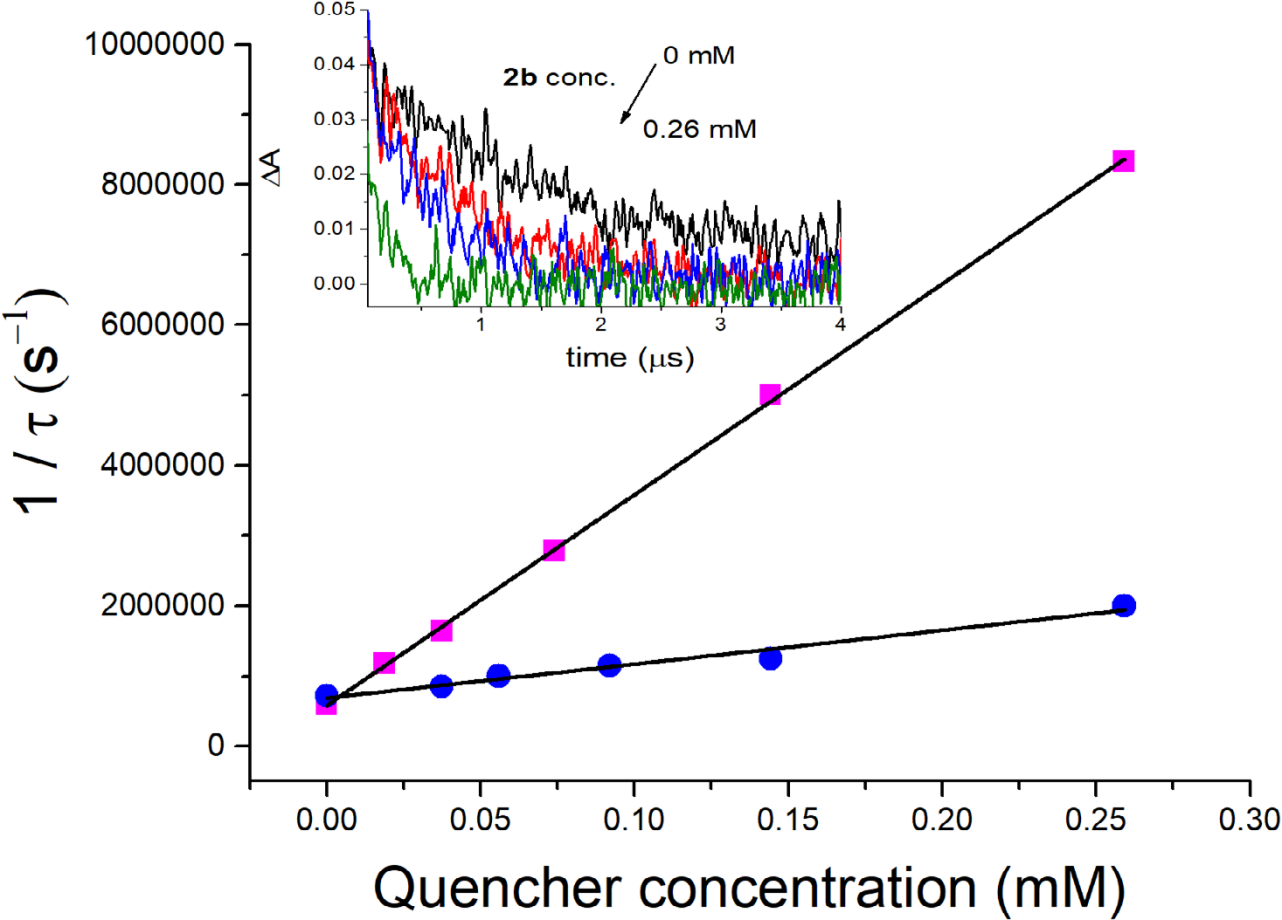
Stern–Volmer plots for the quenching of the hydrated electron by **1b** (blue circles) and **2b** (pink squares). Inset: Decays registered at 720 nm of N_2_‐degassed DMA solutions in water in the presence of increasing amounts of **2b**.

To achieve a higher solubility of the quenchers in water, **1a** and **2a** were deprotected under mild conditions (see Material and Methods section) to give **1b** and **2b**, respectively (Chart 1). Decays at 720 nm were registered for DMA aqueous solution under N_2_ atmosphere in the presence of increasing amounts of the 6‐4PP moieties. The e^‐^_aq_ decay was accelerated in the presence of both quenchers (Fig. 5 and Figure S4). However, bimolecular rate constants of 3 × 10^10^ and 4 × 10^9^ M^‐1^s^‐1^ were obtained from the Stern–Volmer plots (Fig. 5) for quenching by **2b** and **1b**, respectively. This revealed that, as shown above with the fluorescence experiments, the former compound is more susceptible to be reduced.

## Conclusion

Although the reduction potentials (*E*_red_) of **1** and **2** have not been reported in the literature, their ability to act as electron acceptors can be deduced by comparison with structurally related compounds. In the case of **2**, an approximated value of *E*_red_= −0.75 V vs SCE can be considered, it was obtained from the first one‐electron wave of the electrochemical reduction of 2‐pyrimidone in aqueous media (37). For compound **1**, the value can be estimated to be close to that reported for 5,6‐dihydrothymine, *E*_red_= −2.07 V vs SCE (38), or for thymine‐derived oxetanes, *E*_red_= −2.4 V vs SCE. (39) Taking into account of these data, it clearly appears that the pyrimidone chromophore should be more easily reduced than the 5‐hydroxy‐5,6‐dihydrothymine part, which is in full agreement with the spectroscopic results described above. Interestingly, the direction of the electron towards the 3′‐ or 5′‐base of 6‐4PP has not been identified for the real system, and both sites have been proposed as the initial reduced moiety of the photorepair cycle. Recent studies computed the forward and back electron transfer (FET and BET, respectively) from the ^1^FADH^‐^* to the 2‐pyrimidone or 5‐hydroxy‐5,6‐dihydrothymine components and showed that the ratio between FET and BET rates is optimum when the pyrimidone is the electron accepting moiety (24). Indeed, this moiety has a more efficient FET and less favored BET than the 5‐hydroxy‐5,6‐dihydrothymine part. However, experimental results based on the fast BET kinetics obtained by ultrafast spectroscopy seem to be more in accordance with the 5′‐base being the reduced part in the enzymatic process.

It is noteworthy that our study was performed in solution and does not reproduce the exact environment of the photolyase active site where histidines were shown to play a crucial role in the repair process through H‐bonding to the 6‐4PP. These interactions can modify the electron affinity of the two components and control the direction of the electron transfer to this dimeric lesion.

## Supporting information


**Figure S1**. Normalized spectral overlap of emission for phenanthrene alone (red), or in the presence of **2a** (0.42 mM) (blue) and absorption of **2a**.
**Figure S2**. Emission for phenanthrene (8 × 10^‐6^ M, red), **1a** (0.4 × 10^‐3^ M, black) and **2a** (0.4 × 10^‐3^ M, blue) after excitation at 260 nm. Inset: Decay of **2a** registered at 380 nm after excitation at 256 nm.
**Figure S3**. Steady‐state fluorescence spectra of acetonitrile solutions of carbazole (A) and 2‐methoxynaphthalene (B) in the presence of increasing amounts of **2a**, and of carbazole (C) and 2‐methoxynaphthalene (D) with of increasing concentrations of **1a**. λ_exc_=260 nm.
**Figure S4**. Decays registered at 720 nm of N_2_‐degassed DMA solutions in water in the presence of increasing amounts of **2b** (A) or **1b** (B).
**Table S1**. Absorbance and molar absorption coefficient (ε = 260) at 260 nm of **1a, 2a** and photoreductants in acetonitrile.
