# Supplementary material for: Model Studies on the Photoreduction of the 5‐Hydroxy‐5,6‐dihydrothymine and 5‐Methyl‐2‐pyrimidone Moieties of (6‐4) Photoproducts by Photolyase
Source: Photochem Photobiol. 2022 Jan 28;98(3):671–7. doi: 10.1111/php.13592 (PMC9304215; doi:10.1111/php.13592)
Supplement: Supplementary file 1 — Figure S1. Normalized spectral overlap of emission for phenanthrene alone (red), or in the presence of 2a (0.42 mM) (blue) and absorption of 2a. Figure S2. Emission for phenanthrene (8 × 10‐6 M, red), 1a (0.4 × 10‐3 M, black) and 2a (0.4 × 10‐3 M, blue) after excitation at 260 nm. Inset: Decay of 2a registered at 380 nm after excitation at 256 nm. Figure S3. Steady‐state fluorescence spectra of acetonitrile solutions of carbazole (A) and 2‐methoxynaphthalene (B) in the presence of increasing amounts of 2a, and of carbazole (C) and 2‐methoxynaphthalene (D) with of increasing concentrations of 1a. λexc=260 nm. Figure S4. Decays registered at 720 nm of N2‐degassed DMA solutions in water in the presence of increasing amounts of 2b (A) or 1b (B). Table S1. Absorbance and molar absorption coefficient (ε = 260) at 260 nm of 1a, 2a and photoreductants in acetonitrile. [file PHP-98-671-s001.pdf]

# SUPPORTING INFORMATION

# Model Studies on the Photoreduction of the 5-Hydroxy-5,6-dihydrothymine and 5-Methyl-2-pyrimidone Moieties of (6-4) Photoproducts by Photolyase

Gemma M. Rodríguez-Muñiz, Miguel A. Miranda*, Virginie Lhiaubet-Vallet*

Instituto Universitario Mixto de Tecnología Química (UPV-CSIC), Universitat Politècnica de València, Consejo Superior de Investigaciones Científicas, Avda de los Naranjos s/n, 46022 Valencia, Spain

*Corresponding author e-mail:

mmiranda@qim.upv.es (MAM), lvirgini@itq.upv.es (VL-V)

## Index:

**Figure S1.** Normalized spectral overlap of emission for phenanthrene alone (red), or in the presence of **2a** (0.42 mM) (blue) and absorption of **2a** – *Page S1*

**Figure S2.** Emission for phenanthrene (8 × $10^{-6}$ M, red), **1a** (0.4 × $10^{-3}$ M, black) and **2a** (0.4 × $10^{-3}$ M, blue) after excitation at 260 nm. Inset: Decay of **2a** registered at 380 nm after excitation at 256 nm. – *Page S2*

**Figure S3.** Steady-state fluorescence spectra of acetonitrile solutions of carbazole (A) and 2-methoxynaphthalene (B) in the presence of increasing amounts of **2a,** and of carbazole (C) and 2-methoxynaphthalene (D) with of increasing concentrations of **1a.** $\lambda_{exc}$=260 nm – *Page S3*

**Figure S4.** Decays registered at 720 nm of $N_2$-degassed DMA solutions in water in the presence of increasing amounts of **2b** (A) or **1b** (B) – *Page S4*

**Table S1.** Absorbance and molar absorption coefficient ($\varepsilon_{260}$) at 260 nm of photoreductants and **2a** in acetonitrile – *Page S5*

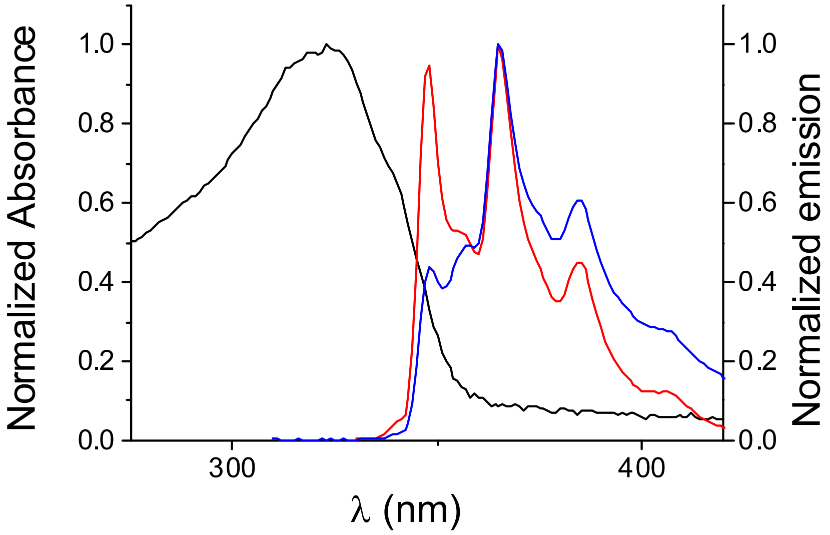


**Figure S1.** Normalized spectral overlap of emission for phenanthrene alone (red), or in the presence of **2a** (0.42 mM) (blue) and absorption of **2a**.

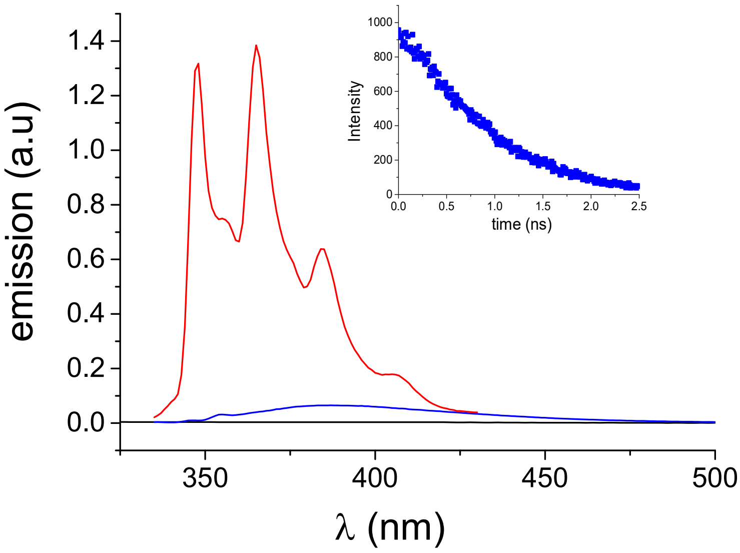


**Figure S2.** Emission for phenanthrene ($8 \times 10^{-6}$ M, red), **1a** ($0.4 \times 10^{-3}$ M, black) and **2a** ($0.4 \times 10^{-3}$ M, blue) after excitation at 260 nm. Inset: Decay of **2a** registered at 380 nm after excitation at 256 nm.

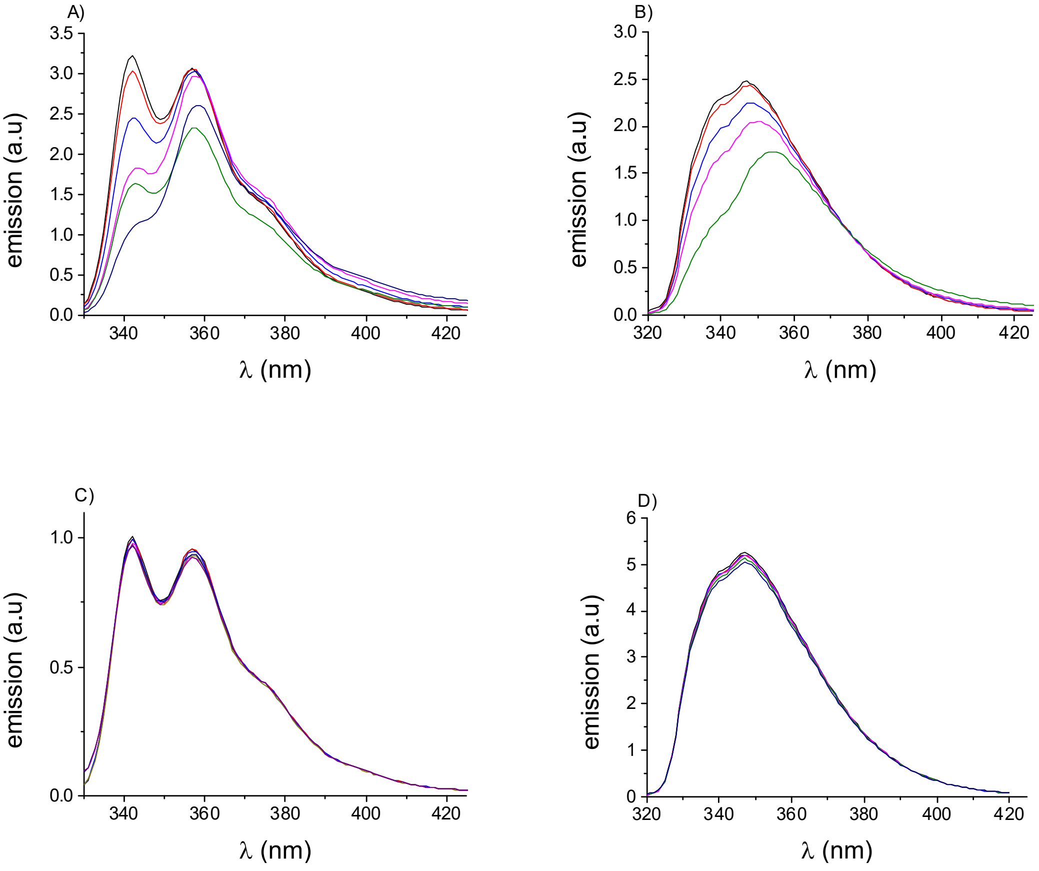


**Figure S3.** Steady-state fluorescence spectra of acetonitrile solutions of carbazole (A) and 2-methoxynaphthalene (B) in the presence of increasing amounts of **2a,** and of carbazole (C) and 2-methoxynaphthalene (D) with increasing concentrations of **1a.** $\lambda_{exc}$=260 nm

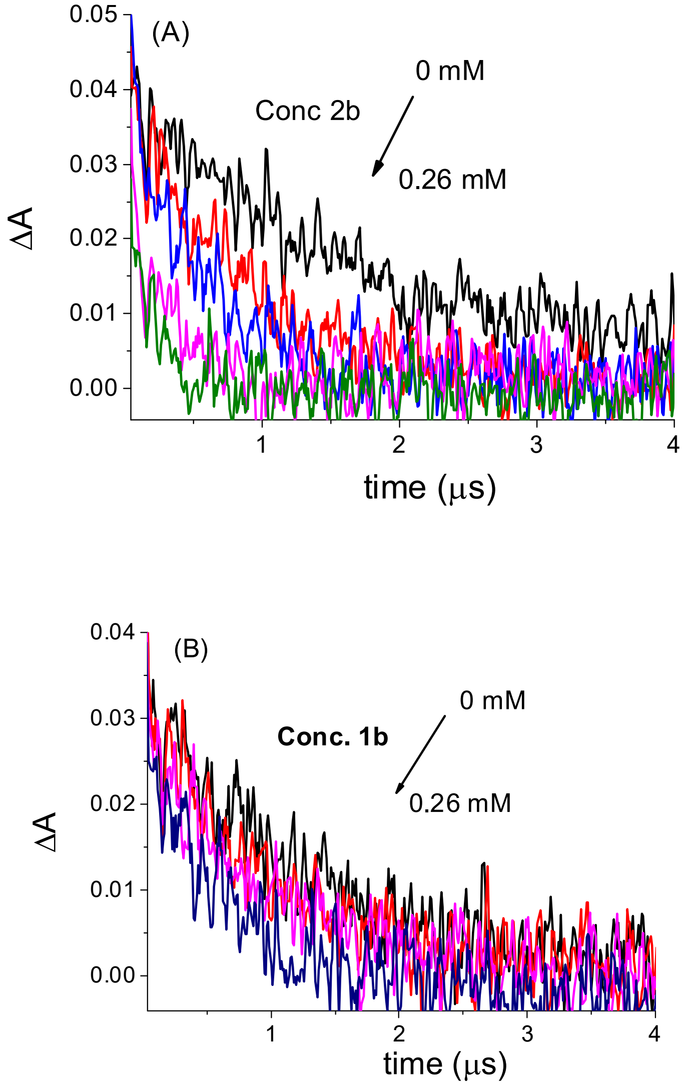


**Figure S4.** Decays registered at 720 nm of $N_2$-degassed DMA solutions in water in the presence of increasing amounts of **2b** (A) or **1b** (B).

**Table S1.** Absorbance ($A_{260}$) and molar absorption coefficient ($\varepsilon_{260}$) at 260 nm of **1a**, **2a** and photoreductants in acetonitrile.

| Compound | Concentration (M) | $A_{260}$ | $\varepsilon_{260}$ ($M^{-1}cm^{-1}$) |
|---|---|---|---|
| **1a** | $4 \times 10^{-4}$ | 0.06 | 150 |
| **2a** | $4 \times 10^{-4}$ | 0.40 | 1000 |
| 2-Methoxynaphthalene | $3.4 \times 10^{-5}$ | 0.13 | 3800 |
| Carbazole | $2.75 \times 10^{-5}$ | 0.10 | 3760 |
| Phenanthrene | $8 \times 10^{-6}$ | 0.12 | 15200 |
